# Origins of Dissociations in the English Past Tense: A Synthetic Brain Imaging Model

**DOI:** 10.3389/fpsyg.2021.688908

**Published:** 2021-07-02

**Authors:** Gert Westermann, Samuel Jones

**Affiliations:** Department of Psychology, Lancaster University, Lancaster, United Kingdom

**Keywords:** English past tense, connectionist modeling, synthetic brain imaging, experience-dependent brain development, verb inflection, verb morphology, neuroconstructivism

## Abstract

Brain imaging studies of English past tense inflection have found dissociations between regular and irregular verbs, but no coherent picture has emerged to explain how these dissociations arise. Here we use synthetic brain imaging on a neural network model to provide a mechanistic account of the origins of such dissociations. The model suggests that dissociations between regional activation patterns in verb inflection emerge in an adult processing system that has been shaped through experience-dependent structural brain development. Although these dissociations appear to be between regular and irregular verbs, they arise in the model from a combination of statistical properties including frequency, relationships to other verbs, and phonological complexity, without a causal role for regularity or semantics. These results are consistent with the notion that all inflections are produced in a single associative mechanism. The model generates predictions about the patterning of active brain regions for different verbs that can be tested in future imaging studies.

## Introduction

The English past tense has, over the past 35 years, taken center stage in the debate on the nature of language and cognitive processing. This is because the past tense is a prototypical “rules-and-exceptions” system, with regular verbs that form their past tense by adding –*ed* to the stem (e.g., *look-looked*), and irregular verbs with past tense forms that range from no change (*hit-hit*) and vowel changes with or without suffixation (*sleep-slept, sing-sang*) to completely idiosyncratic forms (*go-went*). The main question around which this debate has revolved is whether there are separate processing mechanisms for regular and irregular verbs, or if they can be accounted for in a system that produces both regular and irregular forms through a single associative mechanism. This question is important because it has wider implications, for example, for the rule-like nature of grammar (*is rule-like behavior evidence for an underlying mental rule or can it be explained through associative processes?*) and for the question of whether behavioral dissociations imply that the language system has a modular architecture. These questions touch on the very nature of language and cognitive processing, and the English past tense has therefore been called the “drosophila of language processing” (Pinker, 1994): a model system in which such questions can be studied in detail.

Two dominant theories of the nature of inflection processing have emerged. One view, the dual-mechanism or words-and-rules theory (e.g., Pinker, 1991, 1997, 1999; Marcus et al., 1995; Ullman et al., 1997; Clahsen, 1999; Pinker and Ullman, 2002; Ullman, 2004) holds that the processing differences between regular and irregular forms that have been observed in many studies are caused by distinct, qualitatively different underlying mechanisms: A mental symbolic rule for regular forms, and associative storage in the mental lexicon for irregular forms. According to this view grammatical differences are psychologically real in that the mental grammar is used directly in language processing (Clahsen, 1999), so that language processing separates into an associative mental lexicon and a rule-based system (i.e., words-and-rules).

An alternative view argues that all past tense forms are processed in a single associative system in which overlapping representations for regular and irregular forms compete for processing resources (e.g., Bybee and Slobin, 1982; Rumelhart and McClelland, 1986; MacWhinney and Leinbach, 1991; Plunkett and Marchman, 1991, 1993; Marchman, 1993; Joanisse and Seidenberg, 1999; Plunkett and Juola, 1999; McClelland and Patterson, 2002; Westermann and Plunkett, 2007; Westermann and Ruh, 2012; Engelmann et al., 2019). This view is closely tied to implemented connectionist neural network models that have simulated how graded dissociations between different verbs can arise without recourse to modularity and qualitatively different processes. In these systems, apparent dissociations between regular and irregular forms emerge on the basis of the different statistical properties of verbs, such as frequency, phonological complexity, similar sounding verbs with a similar sounding past tense form (i.e., “friends;” e.g., *sing* and *ring*), similar sounding verbs with a different sounding past tense form (i.e., “enemies;” e.g., *sing* and *bring*), or due to reliance on semantic vs. phonological factors.

A large amount of empirical and computational work has aimed to provide evidence for each view [for an overview, see McClelland and Patterson (2002), Pinker and Ullman (2002), and Westermann and Ruh (2012)]. While much of this research has focused on behavioral data from language acquisition and studies involving adults with and without brain damage, a number of brain imaging studies have also revealed brain regions involved in processing different verb inflections. These studies have found differences in neural activation patterns when participants inflected regular and irregular verbs, evidence cited by some researchers as support for a dual mechanism system in which the rule component and the associative mental lexicon are located in different brain regions [e.g., Jaeger et al., 1996; Lavric et al., 2001; Dhond et al., 2003; Sahin et al., 2006; Oh et al., 2011; Bakker et al., 2013; for an overview see Leminen et al. (2019)]. For example, in a seminal study by Jaeger et al. (1996) using positron-emission tomography (PET), participants were asked to generate past tense forms of visually presented monosyllabic verb stems. Jaeger et al. (1996) predicted that the left frontal lobe should be involved in regular processing due to its role in grammatical processing. Likewise, inflection of irregulars was predicted to involve posterior temporal or parietal activity as an index of memory retrieval. Results showed that although many brain regions were activated equally by all verbs, production of regulars selectively activated left dorsolateral prefrontal cortex and left anterior cingulate cortex. Irregulars, meanwhile, prompted higher overall activation and involved occipital visual processing areas. These systematic differences between both verb types were interpreted by the authors as strong evidence for the dual-mechanism account of inflection. Similar claims were made by Lavric et al. (2001) in an ERP study of covert past tense production. These authors found differences between regular and irregular past tense forms in a time window from 288 to 321 ms after visual presentation of the verb stem, and source localization indicated higher activation during this time window for regulars in right prefrontal and temporal areas and higher activation for irregulars in the left temporal area and the anterior cingulate cortex.

In another study using magnetoencephalography (MEG), Dhond et al. (2003) asked participants to covertly generate past tense forms of visually presented verb stems. Dhond et al. also found that generation of regulars and irregulars activated many brain areas in common, but that processing of regulars led to greater activation in left inferior prefrontal areas (Broca's area), and processing of irregulars preferentially activated left occipitotemporal cortex as well as right dorsolateral prefrontal cortex. These results were interpreted as indicating that regulars activated rule-based grammar regions and irregulars activated areas involved in the associative retrieval of forms, corresponding directly to the dual-mechanism theory. Different results were found in an fMRI study of covert past tense and plural production (Sahin et al., 2006) in which Broca's area was activated equally by regular and irregular verbs. Irregulars activated the anterior cingulate and supplementary motor area more than regulars, whereas regulars led to greater activation in some subcortical structures. Overall there was greater activation for irregulars. These results were interpreted within the dual-mechanism framework by suggesting that activation differences between regulars and irregulars were evidence for separate mechanisms and, therefore, against a single mechanism of inflection. Specifically, it was argued that Broca's area was involved in inflection processing, and that greater activation associated with irregulars indicated blocking of the application of the rule by a retrieved irregular form.

However, the results of these and other studies have been controversial. One problem is that specific methodological choices can strongly affect results. For example, because of the low temporal resolution of PET, Jaeger et al. (1996) used a block design in which all regular verbs and all irregular verbs were presented together. However, this design introduces the confound that participants could develop response strategies for regular but not for irregular verbs, suggesting that differences between both verb types should be found independently of the nature of the underlying processing mechanisms (Seidenberg and Hoeffner, 1998). Furthermore there have been inconsistencies between studies in the brain areas that were activated by different verbs [see also Table 1 in Desai et al. (2006)]. For example, Broca's area was activated selectively by regulars in one study (Dhond et al., 2003) which led the authors to argue that it is responsible for rule-based processing, but it was active equally for regular and irregular verbs in another (Sahin et al., 2006). Likewise, greater activation of the anterior cingulate cortex was found for regulars in one study (Jaeger et al., 1996) and for irregulars in others (Lavric et al., 2001; Sahin et al., 2006).

Several other imaging studies have investigated the possibility that the observed activation differences between regular and irregular verbs are due to the different statistical properties of verbs and not to separate underlying mechanisms. For example, an fMRI study in which participants covertly produced the past tense of auditorily presented stems (Joanisse and Seidenberg, 2005) found that regulars and irregulars activated common areas in both hemispheres, but that regulars, as well as irregulars that were phonologically similar to regulars (e.g., *burnt, slept*), additionally activated the inferior frontal gyrus bilaterally. In this study, irregulars did not activate any area more than regulars. Dissociations between verbs were thus argued to arise from the phonological properties of verbs instead of their regularity. In a similar fMRI study, Desai et al. (2006) also found widespread overlapping activation, including in Broca's area, for all verbs, and greater activation for regulars in the left dorsal superior temporal gyrus, involving the primary auditory areas and the planum temporale. This study also found regions of greater activation for irregulars compared with regulars (inferior frontal, precentral cortex and parietal cortex bilaterally). When the authors matched a subset of their verb set for phonological complexity of the past tense form, they found that no regions were activated more for regulars than for irregulars. Desai et al. (2006) explained the widespread activation of brain regions for irregular verbs in terms of higher demands on attention, working memory, and response selection for generating the past tense forms of these verbs. The fact that both regular and irregular production activated Broca's area was seen as contradicting the dual-mechanism account which assumes that regular, but nor irregular forms are generated through a mental grammar instantiated in Broca's area (Ullman et al., 1997). Greater activation in auditory areas for regulars was explained with regular forms being phonologically more complex than irregular forms (Burzio, 2002; Bird et al., 2003). Therefore, despite double dissociations between regular and irregular verbs these results were interpreted as evidence for a single-mechanism view of inflection processing.

In summary, previous imaging studies, despite each reporting single or double dissociations between regular and irregular verbs, have not provided a coherent picture of the brain areas involved in processing the English past tense: First, the activated regions for specific verb types differed considerably between studies; and second, the nature of the dissociations differed between studies. One study (Joanisse and Seidenberg, 2005) reported activation of distinct brain regions for regulars but not irregulars, another (Desai et al., 2006) reported the opposite pattern with distinct regions active for irregulars but not for regulars when verbs were matched phonologically, and other studies (Jaeger et al., 1996; Dhond et al., 2003; Sahin et al., 2006; Oh et al., 2011) reported a double dissociation with some regions more active for regulars and others more active for irregulars (although these regions differed in each case). These inconsistent patterns of activation have made it difficult to sufficiently constrain the theories of inflection for (or against) which they were meant to provide evidence. For example, involvement of Broca's area in the inflection of both regular and irregular verbs has been claimed to provide evidence both for (Sahin et al., 2006) and against (Desai et al., 2006) dual-mechanism views of inflection.

One possible explanation for the inconsistency in observed activation patterns in the discussed neuroimaging studies is that statistical factors and not grammatical class determine how a verb is processed, and that these factors differed between the specific verb stimuli used in existing studies. In each study, regular and irregular verbs were matched on certain factors, but the choice of factors had little theoretical foundation and differed greatly between studies. Jaeger et al. (1996) matched stem and past tense frequencies (albeit based on a word list that did not distinguish between nouns and verbs and therefore overestimated regular stem frequencies), Lavric et al. (2001) and Dhond et al. (2003) matched word frequency and letter length, Sahin et al. (2006) matched past and stem cluster frequency and syllable length and aimed for phonological similarity, Oh et al. (2011) matched phonological complexity and past tense frequency, and Joanisse and Seidenberg (2005) matched past tense frequency, imageability, and concreteness. The most careful matching was done by Desai et al. (2006), with past tense frequency, friend-enemy ratio, stem letter length, and stem and past tense syllable length all taken into account, in addition to a sub-group of verbs being further matched on number of phonemes and past tense syllable structure. However, which of these factors affect processing, and in what way, remains an open question. It is therefore also unclear whether a processing system that is sensitive to the statistical properties of verbs would give rise to the observed dissociations in active brain regions.

One approach to answering these questions is to consider how the adult language processing system is shaped through development. Adult psycholinguistics traditionally pays little heed to the mechanisms of language development although a better understanding of developmental trajectories could inform the nature of the adult processing system. Taking this perspective, in this paper we train an artificial neural network model on English past tense inflection (Westermann and Ruh, 2012), adopting a neuroconstructivist developmental process in which the architecture of the adult inflection processing system emerges through an interaction between experience-dependent structural development and experiences with verbs that have specific statistical properties. We then use what has been called “synthetic brain imaging” (e.g., Arbib et al., 1994; Tagamets and Horwitz, 1998, Cangelosi and Parisi, 2004, Horwitz et al., 1999; Arbib et al., 2000; Thomas et al., 2012) to analyze activation patterns across different parts of the model and show that such a system displays visible processing differences between regular and irregular verbs without relying on built-in dissociable processing modules. Finally, we investigate which statistical properties account for the observed dissociations, generating predictions for behavioral and imaging studies.

The computational model used in the current paper was developed by Westermann and Ruh (2012) for modeling behavioral aspects of the acquisition and adult processing of the English past tense. This model displayed a realistic acquisition profile, adult-like non-word generalization, and selective breakdown after damage to parts of the network. The model is based on the neuroconstructivist framework (Quartz and Sejnowski, 1997; Mareschal et al., 2007; Westermann et al., 2007), which stresses the importance of experience-dependent structural brain development in shaping an adult processing system that is specifically adapted to the learning task. There is overwhelming evidence that experience shapes the brain during cognitive development (e.g., Quartz and Sejnowski, 1997; Quartz, 1999; Casey et al., 2000, 2005; Johnson, 2001; Johnson and Munakata, 2005; Nelson et al., 2006; Mareschal et al., 2007; Westermann et al., 2007; Stiles, 2009; Bick and Nelson, 2017), and that differences in adult brain structures can at least partly be explained by a developmental process by which the brain adapts to the specific aspects of the tasks being learned. For example, one study involving Chinese-speaking adults and English-speaking adults living in the United States reported specific differences in the size of frontal, temporal and parietal cortical substrates between these groups (Kochunov et al., 2003). This structural difference was interpreted by the authors as an outcome of the different orthographic, phonetic and semantic characteristics of Chinese and English, which impacted experience-dependent brain development. Likewise, structural brain changes have been observed when learning a second language [see Li et al. (2014), for a review] and for bilinguals [see Bialystok (2017), for review]. For example, native Japanese speakers trained on learning English words for 16 weeks showed an increased density of gray and white matter in the right IFG, but a control group did not show these changes (Hosoda et al., 2013). Anatomical change in this study correlated positively with the participants' knowledge of English vocabulary. Other studies have begun to address systematic cross-linguistic variation in the neural structures supporting language processing (Chen et al., 2009; Mei et al., 2015) and more broadly have asked how specific experiences affect brain organization in members of different cultures (Park and Huang, 2010). Here, along similar lines, we argue that the specific processing demands of the English inflection system will lead to brain structures that are adapted to these demands through experience-dependent development. From this perspective, the specific dissociations between brain activation patterns observed in the adult language system are the outcome of experience-dependent structural development under the task demands of learning verb inflections with their characteristic distribution and statistical properties. This view is in contrast to a modular view of language processing according to which functionally specialized modules implement qualitatively different mental processes (e.g., Pinker, 1994).

Translating these ideas into a computational model, the artificial neural network developed by Westermann and Ruh (2012) integrates structural changes that mimic, on an abstract level, the experience-dependent development of cortical regions through childhood, allowing for the adaptation of its neural circuits to the specific demands of learning to inflect a large set of English verbs. It should be noted, however, that this relatively simple model, despite integrating aspects of neural development, is not a computational neuroscience model that aims to account for the formation of biological synapses or the internal processes of biological neurons, or to mimic the specific aspects of experience-dependent brain development. Connectionist models are usually conceptualized as higher-level models that are based on an abstract and simplified view of neural processing in the brain (e.g., Rumelhart, 1989): Interactions between simple processing units to generate complex behavior; learning of associations by adapting the efficacy of transmission between processing units; and the ability to extract statistical structures from the environment. As high-level models, units (“neurons”) in connectionist models are not assumed to correspond to biological neurons on a one-to-one basis but instead to large ensembles of biological neurons (e.g., O'Reilly and Munakata, 2000). Nevertheless, the model is grounded in the assumptions that first, task-driven structural adaptation during learning qualitatively changes the learning process compared with learning in a fixed structure (Quartz, 1993; Quartz and Sejnowski, 1997; Westermann and Ruh, 2012; Westermann, 2016), and second, that it shapes the functional structure of the final system (Mareschal et al., 2007; Shultz et al., 2007) so that the adult system can best be understood as an outcome of such a structural developmental process. In building the model, therefore, we were interested in a principled mechanistic account of how a processing system that is sensitive to the statistical properties of verbs to which it is exposed while undergoing a structural developmental process, gives rise to the dissociations between activation patterns for different verbs that are observed in adult neuroimaging studies.

Synthetic brain imaging (Arbib et al., 1994, 2000; Tagamets and Horwitz, 1998; Horwitz et al., 1999) applies the idea of brain imaging—comparing brain region activation profiles between different test conditions to gain insights into underlying processing mechanisms—to artificial neural networks. In a structured neural network, different stimuli will generate specific activation patterns in different network components and, like in brain imaging, these patterns can be compared between conditions. Although synthetic brain imaging is still in initial stages of exploration, several results have been reported in modeling language processing. For example, one study showed how differential activation patterns for nouns and verbs arose in evolved agent-based networks (Cangelosi and Parisi, 2004): Whereas nouns activated preferentially sensory processing areas of the networks, verbs activated multisensory integration areas more broadly. These activation patterns were compared with brain imaging data showing that nouns activate more posterior brain areas whereas verbs also activate anterior motor areas. In another study, synthetic brain imaging was used in a model of sentence comprehension (Just et al., 1999) and accounted for fMRI data on brain regions involved in processing sentences of different complexities. A third study used synthetic brain imaging and lesioning to investigate whether impairment after brain damage and neuroimaging predict the same patterns of functional specialization (Thomas et al., 2012).

In deciding how to measure the synthetic analog to the fMRI BOLD signal it is important to consider which aspect of neural processing is reflected by BOLD. Current understanding is that the BOLD signal in fMRI does not measure neural activity (i.e., spike potentials) but rather the local field potential (LFP), which reflects the summation of post-synaptic potentials (Logothetis et al., 2001; Norris, 2006). This view would indicate that the closest correlate in a connectionist model to the BOLD signal is the incoming activation into a group of units, that is, the activation flowing through a pathway to this set of units. While it is beyond the scope of this report to explain in detail the many different activation patterns that have been observed in neuroimaging studies of the past tense, we aim to show how differential activation patterns can be generated in a single-mechanism system that is shaped through interactions between the statistical structure of the environment and experience-dependent brain development. In doing so we will account for some empirical results in detail and generate predictions for future neuroimaging studies.

There are a number of reasons why synthetic brain imaging using neural networks can inform theory building and help generate predictions for assessment in studies using real brain imaging. First, in neural networks, the experimenter has full control over the studied process. In imaging studies of inflection processing, the large number of active brain areas suggests that it is difficult to find a baseline condition that differs from the experimental condition only in the inflection process. For example, Desai et al. (2006) reported that the baseline task of reading verbs activated some brain regions that were not active when the verbs were inflected. In a model of verb inflection that takes a verb stem as input and produces its past tense as output, the inflection process can be isolated effectively. It is therefore not necessary to establish a baseline condition (such as reading a verb without inflecting it) and subtracting this baseline activation from the observed activation patterns in the inflection task. Second, a computational model allows for the precise analysis of what factors affect differential activation of network components in a much larger set of verbs than those typically used in neuroimaging studies, where small sets of verbs have to be matched for statistical factors. Third, the language experience that has shaped the computational model toward its final structure is precisely known and is under the control of the modeler. This allows for a better characterization of the statistical factors that underpin emerging dissociations.

## Materials and Methods

### The Model

**Figure 1:**
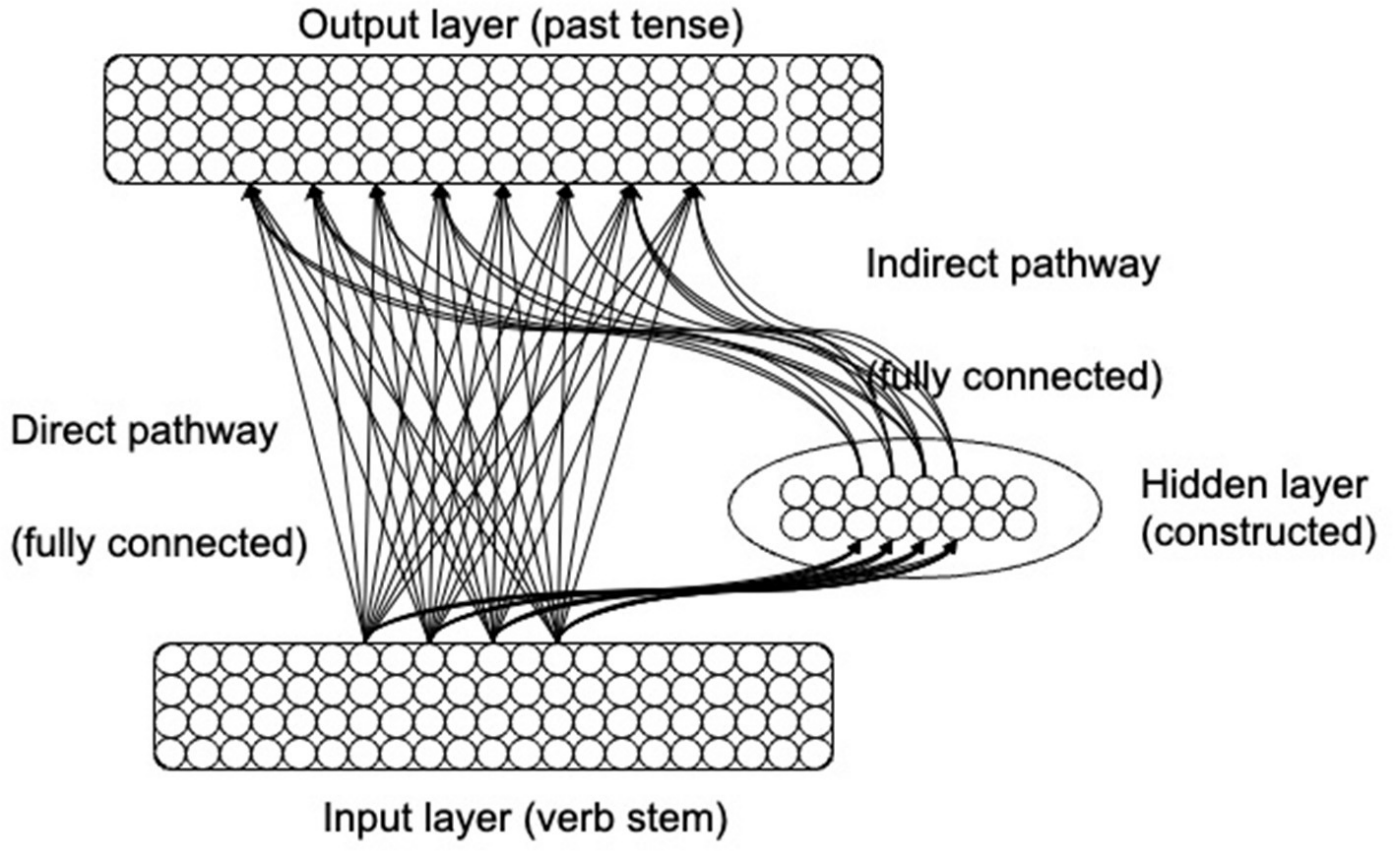
The architecture of the neuroconstructivist past tense model.

The neuroconstructivist neural network model (NCM; Westermann and Ruh, 2012) (Figure 1) starts out with a minimal architecture in which the input and output layers are fully connected. In a process of experience-dependent structural development, the hidden layer gradually expands to enable the past tense inflection task to be learned. The “adult” architecture of the model is therefore an outcome of, and optimally adapted to, the specific learning task.

In (Westermann and Ruh, 2012), the NCM was presented with phonological representations of verb stems and had the task of producing the corresponding past tense forms. Hidden layer units had a Gaussian (i.e., bell-shaped) activation function. Units of this type become active for a subset of similar-sounding verbs, forming a receptive field for a region of the phonological input space. Gaussian units are activated when an input (i.e., a verb stem) falls within their receptive field, and the closer the input is to the center of the receptive field the higher is the activation of the unit. In the NCM, lateral inhibition in the hidden layer was simulated by suppressing activation of all but the most active hidden unit. The position of the receptive field of this unit was adjusted at each presentation to move a small step toward the position of the current input. Receptive field sizes were also adapted to increase for fields that responded to a range of different verbs. Each hidden unit kept a local error counter to which the network's output error was added when the hidden unit was active.

The model attempted to learn the inflection task in the initial minimal architecture, and structural change occurred when the current structure no longer allowed for improvement in performance: When the average error over 10,000 verbs was no lower than for the previous 30,000 verbs, three new hidden unit receptive fields were inserted at the position of the existing hidden unit which had the highest local error, and their weights to the output layer were initialized randomly. In this process, a hidden unit whose activation leads to a high output error will become the preferred location for the insertion of new units. Because a high local error is usually caused by one hidden unit being responsible for too many input patterns with conflicting input-output transformations (e.g., *sink-sank* and *blink-blinked*), the insertion of additional resources led to a more fine-grained covering of the input space in those areas where similar sounding verbs have different past tense forms. As a consequence, the hidden units were “quasi-localist” [see Westermann and Ruh (2012)]: different units became responsive to between 1 and 136 verbs, with the degree of granularity an outcome of the task demands of the past tense inflection task. Note that this is different from purely localist “lexical entry units” (e.g., Joanisse and Seidenberg, 1999) where each unit is activated by exactly one verb.

Regressive events in the model were implemented by pruning hidden units that were not activated for 30,000 verb presentations [For further details of the implementation see Westermann and Ruh (2012)]. Together, these mechanisms led to a process in which the structure of the model—number, size and location of hidden unit receptive fields as well as the weight patterns in both the direct and indirect pathways—was a direct outcome of the experience with the environment of the English past tense, with its different verbs with specific inflections, phonological properties, similarity clusters, and frequencies. This way of developing the model is in contrast both to the more common static models in which only the weights but not the model structure are adapted, and to models in which change proceeds along a maturational timetable independent from environmental input (e.g., Elman, 1993). Indeed, whereas the developing model was shown to account for a wide range of data on acquisition, adult generalization, and selective impairment after brain damage, an equivalent static model did not account for many of these data (Westermann and Ruh, 2012).

This “neuroconstructivist” type of model also corresponds most closely to current views of experience dependent brain development in which new abilities become manifest in developing brain structures that are adapted to the demands of a specific ability [see also Shultz et al. (2007)]. In the past tense model, more structure (i.e., hidden units and their connections) was allocated for forms that were “harder” to learn because of the statistical properties of the verb set.

### Corpus

The NCM was trained on a set of 1,271 mono- and bisyllabic English verbs extracted from the CELEX database [Baayen et al., 1995; full training details are provided in Westermann and Ruh (2012)]. Of these verbs, 111 (i.e., 8.73% of types, 46.00% of tokens) were irregular. During training, verbs were drawn from this corpus on the basis of their past tense frequencies. The phonemes of each verb were inserted into a consonant-vowel template of the form xCCCVCC for each syllable [where x indicates if the syllable was stressed (1) or not (0)]. Individual phonemes were encoded by phonetic feature vectors, following the binary version of the PatPho coding scheme (Li and MacWhinney, 2002) which requires six features per vowel and seven features per consonant. The presence or absence of a feature was encoded by a value of 1 or −1, respectively, and all features for an empty phoneme slot were set to 0. The stem of a verb was encoded by 84 bits and the past tense form had an additional VC suffix (13 bits).

### Training

Five networks were trained on 20 m verb tokens each. Verbs were presented randomly according to their past tense frequencies. Weights were updated after the presentation of each verb (online learning) using the perceptron learning rule (Rosenblatt, 1958). For earlier work on this model see Westermann and Ruh (2009).

### Synthetic Brain Imaging Analysis

Synthetic brain imaging (SBI) in the models was performed by measuring the activation flowing through the direct (input-output) and indirect (hidden-output) pathways for each verb. Activation in the direct pathway was computed as the summed absolute activation flowing through the input-output connections:

$$\begin{array}{l} \underset{o}{\sum }\underset{i}{\sum }|w_{oi}a_{i}| \end{array}$$

where *o* are output units, *i* input units, *w*_*oi*_ the weight of the connection between input unit *i* and output unit *o*, and *a*_*i*_ the activation of input unit *i*. Likewise, activation in the indirect pathway was computed as the absolute activation flowing through the hidden-output connections as:

$$\begin{array}{l} \underset{o}{\sum }|w_{oh}a_{h}| \end{array}$$

where *w*_*oh*_ is the connection weight from the active hidden unit *h* to output unit *o* and *a*_*h*_ the activation of hidden unit *h*. Total activation was computed as the sum of the activation in the two pathways.

## Results

All models reached 100% accuracy on average after exposure to 16.8 million verbs, with an average number of 361 hidden units (range = 355–370). Since performance across networks was highly comparable, detailed results from a randomly sampled network will be reported unless otherwise specified.

### Emerging Double Dissociation Between Regulars and Irregulars

**Figure 2:**
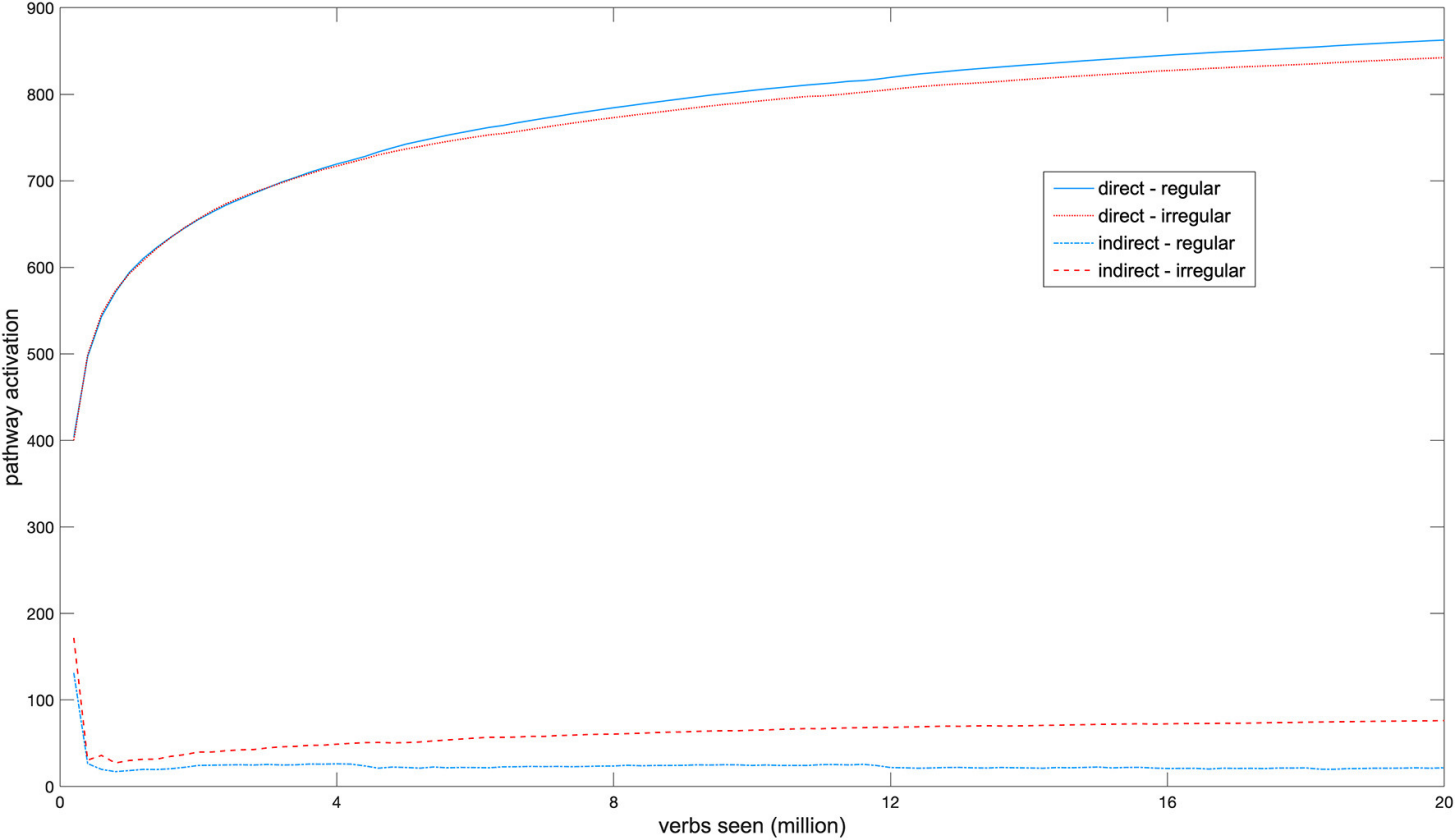
Development of the activation profiles of regular and irregular verbs in both network pathways.

Figure 2 shows a longitudinal developmental SBI activation profile of the two network pathways. Early in development each pathway was activated equally strongly by regular and irregular verbs. With development, activation in the direct pathway increased and separated between regular and irregular verbs, with regulars producing on average higher activation in this pathway than irregulars (mean activation at the end of training by regulars: *M* = 864.3, *SD* = 111.9; by irregulars: *M* = 844.1, *SD* = 91.5; Mann-Whitney *U*-test, *z* = −2.19, *p* = 0.029, mean rank for regulars = 642.97; and for irregulars = 563.21). Overall activation in the indirect pathway initially decreased because activation in this pathway interfered with learning the task due to the insufficient number of hidden units. Throughout the rest of development, mean activation differences between regular and irregular verbs then continued to increase (mean activation at the end of training by irregulars: *M* = 75.5, *SD* = 59.1; by regulars: *M* = 21.4, *SD* = 13.8; Mann-Whitney *U*-test, *z* = −14.534, *p* < 0.001, mean rank for regulars = 589.7, and for irregulars = 1119.7). This double dissociation between regular and irregular verbs emerged in the model without any functional pre-specification of either pathway and without explicit encoding of regularity, solely on the basis of the different task demands of producing the past tenses of different verbs.

Although to our knowledge there have not yet been developmental brain imaging studies of verb inflection, a developmental fMRI study of word production (Brown et al., 2005) found an increase in activation in some cortical areas and a decrease in others across age, with significant differences between age groups even when overt task performance was equal. The network for which results are displayed in Figure 2 reached 100% correct performance in the inflection task after 16.0 million verb tokens, and it is interesting to observe that activation in the direct pathway likewise continued to increase after this point without a change in overt performance.

The fact that a double dissociation in regional activation patterns between regular and irregular verbs emerges in the NCM contradicts the argument that differential activation of brain regions for each verb type necessarily indicates an underlying qualitative processing difference between regular and irregular forms (e.g., Jaeger et al., 1996; Beretta et al., 2003; Sahin et al., 2006). In the “adult” NCM all past tense forms are generated through a single associative mechanism, but dissociations arise on the basis of statistical and distributional differences between verbs that have become manifest in the network's architecture during development. The developing hidden layer enables the model to allocate additional processing resources for verbs whose inflections are hard to learn in the direct pathway alone, as structure is added to this layer when learning no longer improves. The fact that the indirect pathway is activated more by irregular verbs is compatible with the “ease-of-processing” account of functional specialization in past tense processing (Westermann and Ruh, 2012). This account states that on average, irregular forms are harder to learn and process than regulars. An irregular is harder to process than a regular, however, not by virtue of its irregularity, which is a grammatical property of an individual verb, but instead as the result of a combination of statistical and distributional factors such as relative frequency and numbers of friends and enemies, which are statistical properties that arise from the verb corpus as a whole (Westermann and Ruh, 2012). The origin of the emergent dissociations is, therefore, the differential ease of processing of verbs and not their grammatical class.

### Double Dissociations Between Mean Activation Values Mask Distributional Differences

**Figure 3:**
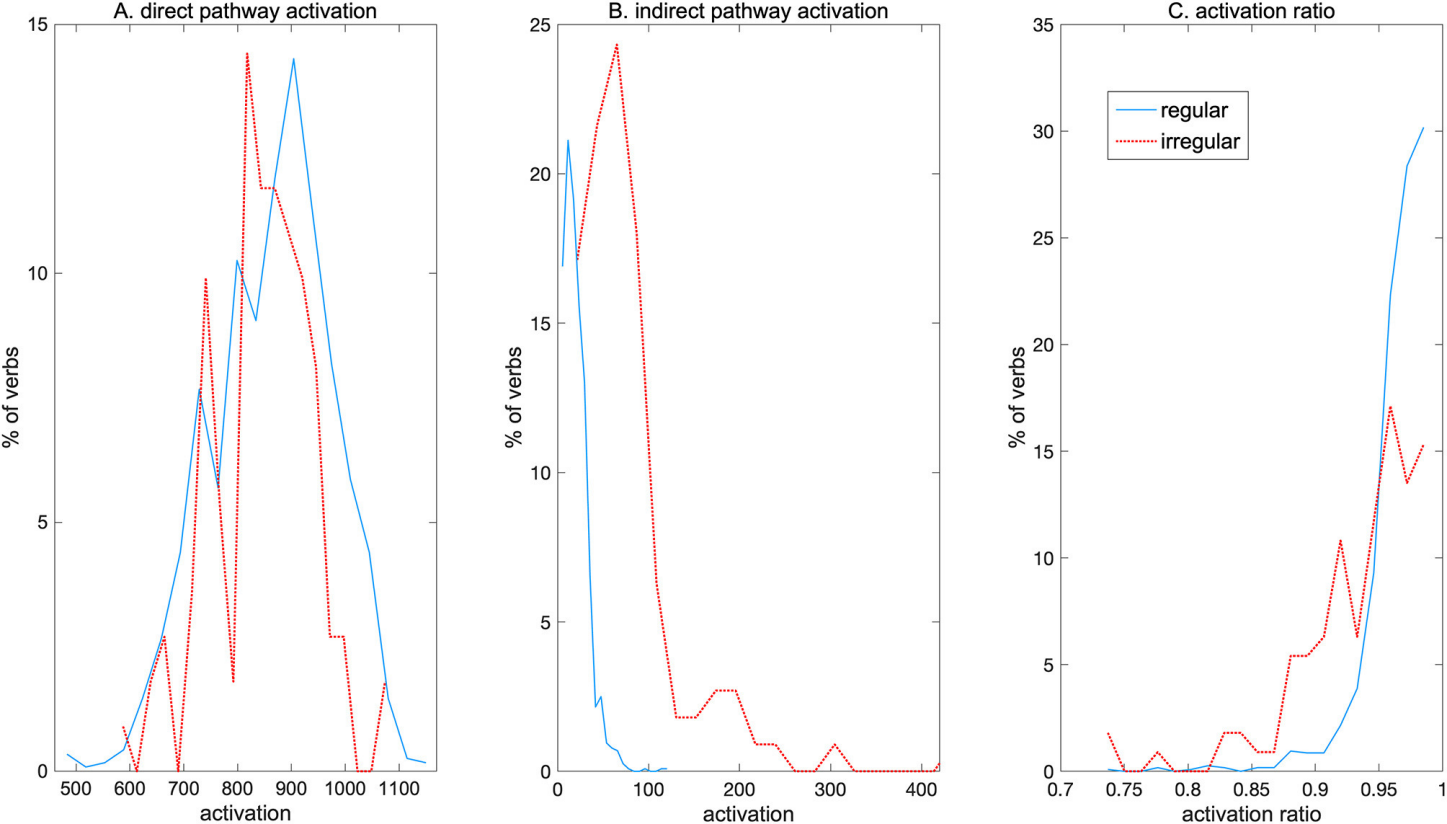
Distribution of path activations by regular and irregular verbs. **(A)** Direct pathway activation. **(B)** Indirect pathway activation. **(C)** Activation ratio.

More detailed analysis of the NCM further revealed that, despite the observed double dissociation between regular and irregular verbs, each verb activated both pathways, albeit to different degrees. Figure 3 shows the distribution of regular and irregular verbs activating each pathway. In the direct pathway (Figure 3A) the spread of activations is similar for regular and irregular verbs, with the highest activations resulting from regulars. In contrast, in the indirect pathway a higher proportion of irregulars than regulars were strongly activated, and most regulars only led to weak activation in this pathway (Figure 3B). Figure 3C shows the *activation ratio* which was computed as:

$$\begin{array}{l} \frac{direct\;pathway\;activation}{direct\;pathway\;activation\;+\;indirect\;pathway\;activation} \end{array}$$

where activations in both pathways were scaled to a maximum value of 1. A ratio of >0.5 indicates that a specific verb activates the direct pathway relatively more than the indirect pathway. The figure shows that regulars as well as irregulars activated the direct pathway more than the indirect pathway, but regulars tended to have a higher activation ratio than irregulars. Nevertheless, some irregulars as well were produced almost solely through the direct pathway, with activation ratio near 1.0. These results indicate that although there is an apparent global specialization of the direct pathway for regular verbs and of the indirect pathway for irregulars as revealed by the observed double dissociation, this is an outcome of complex overlapping activation patterns for individual regular and irregular forms throughout the network.

### Greater Regular Activation Is Due to Greater Phonological Complexity of Regulars

We further modeled more specific results from Desai et al.'s (2006) fMRI study. Desai et al. (2006) argued that a higher activation for regular verbs in some cortical regions was the consequence of the higher phonological complexity of the regular verbs used in their experiment. To test this claim they analyzed a subset of their verbs in which regulars and irregulars were matched for phonological complexity. As predicted, they found that for this matched set there was no brain region more active for regular verbs. We simulated this result by comparing the activation profiles in the model for all verbs with those for the matched subset of Desai et al. (2006). Of the 80 verbs in the subset, two irregulars were not in the network's training corpus (*break, cost*). These word's matched regular partners (*stay, guess*) were also removed from the test set, and the NCM was tested on the remaining 76 matched verbs. For this matched subset, as in Desai et al.'s (2006) study, no area was now more active for regulars. Whereas with the full verb set the average direct pathway activation was higher for regulars than for irregulars (see section Emerging Double Dissociation Between Regulars and Irregulars), the matched subset showed the opposite pattern, with irregulars (*M* = 853.2, *SD* = 99.1) now on average activating the direct pathway more than regulars (*M* = 798.8, *SD* = 92.8; Mann-Whitney *U*-test, *z* = −2.68, *p* = 0.007, mean rank for regulars = 31.71; and for irregulars = 45.29). As in the full set of verbs, indirect pathway activation was higher for irregulars in the matched subset (irregular activation *M* = 58.9, *SD* = 31.3; regular activation *M* = 31.0, *SD* = 20.0; Mann-Whitney *U*-test, *z* = −4.21*, p* < 0.001, mean rank for regulars: 27.84, and for irregulars: 49.16).

Although Desai et al. (2006) found that with the phonologically matched subset some areas were activated more for irregulars than had been for the non-matched set (i.e., the precentral gyrus and left anterior cingulate gyrus), the region previously more active for regulars later showed no difference between regulars and irregulars. While in the NCM this area (i.e., the direct pathway) was now more active for irregulars than for regulars, the model accounted for Desai et al.'s (2006) main result of the disappearance of higher activation for regulars within the processing system when phonological complexity was controlled.

The NCM further provided a more general evaluation of the role of phonological complexity in observed regular-irregular dissociations. Whereas in experimental neuroimaging the effect of phonological complexity can only be controlled for by using matched subsets of verbs, in the NCM the same can be achieved by dividing the total activation in the direct pathway by the number of active input units. This is because in the distributed phonological representation of verbs, higher phonological complexity, here defined as number of phonemes or number of syllables, corresponds to more input units being active. Dividing the direct pathway activation by the number of active input units therefore normalizes this activation (Note that this is not necessary for the indirect pathway because only one hidden unit is active for each verb). Whereas, non-normalized activation in the direct pathway was higher for regulars than for irregulars, activation normalized for complexity was conversely smaller for regulars (*M* = 30.6, *SD* = 8.4) than for irregulars (*M* = 36.0, *SD* = 7.3; Mann-Whitney *U*-test, *z* = −6.82*, p* < 0.001, mean rank for regulars = 614.28, and for irregulars = 862.95), providing further evidence that systematic differences in phonological complexity can lead to regular-irregular dissociations.

### Origins of dissociations

What, then, are the origins of the dissociations found in neuroimaging studies? The “easiness” view of past tense processing suggests that different statistical characteristics of verbs affect their ease of processing, and hence their activation profile, irrespective of whether they are regular or irregular. By using synthetic brain imaging we are able to investigate precisely which statistical factors are involved, as the model is tested on a large set of verbs in which these factors vary considerably. To do this, we characterized each verb along a range of factors that were accessible to the model during training: Past tense frequency, presence of a stem final alveolar consonant, phonological complexity, and number of friends and enemies within the training corpus (Note that a “friend” was defined as a verb with the same stem rime and the same past tense rime, e.g., *sing-sang* and *ring-rang*, and an “enemy” was defined as a verb with the same stem rime but with different past tense rime, e.g., *sing-sang* and *bring-brought*).

Table 1 shows that frequency, friend/enemy measures, and complexity correlate significantly with activation ratio (i.e., direct activation divided by total activation; see Formula 3). These correlations indicate that phonologically complex, low-frequency verbs with an advantageous neighborhood (i.e., many friends, few enemies) tend to activate the direct pathway relatively more strongly (i.e., lead to a higher activation ratio), while the indirect pathway is activated relatively more for frequent verbs with an unfavorable neighborhood.

**Table 1:** Correlations between the statistical properties of verbs and their activation ratio.

**Correlation with activation ratio**	**Past tense frequency**	**Friends**	**Enemies**	**Phonological complexity**
*r*	−0.696	0.226	−0.434	0.245

*All correlations p < 0.001*.

To examine which statistical factors contributed to activation differences in each pathway, we entered these factors as independent variables into multi-level regression models across all networks, with pathway activation as the dependent variable and verb and network as random effects. All predictors were zero centered and scaled to *SD* = 1. Indirect pathway activation was most strongly predicted by past tense frequency (β = 16.77), enemies (β = 5.64), phonological complexity (β = −3.64), and friends (β = −2.86; all *p* < 0.001), with this model explaining 50% of the variance in pathway activation (*R*^2^ = 0.498). For direct pathway activation, the only significant contributing factors were phonological complexity (β = 38.78) and friends (β = 26.43; both *p* < 0.001), with this model accounting for only 19% of the variance in pathway activation (*R*^2^ = 0.189).

The picture emerging from these results is, therefore, slightly more complex than directly linking activation in the indirect pathway with low ease of processing. Although indirect pathway activation is predicted by a high number of enemies and low number of friends—both factors that would be expected to make processing harder—it is also predicted by high frequency, which would be expected to make processing easier. The reason for this counterintuitive result is that verbs that are frequently encountered by the model will lead to the accumulation of many small errors on hidden units (instead of fewer but larger errors for harder verbs) so that in the experience-dependent development of the network's structure new units will also be inserted in those regions of the input space. Importantly, the neuroconstructivist view of past tense processing therefore predicts that the same brain regions should be shared by the processing of frequent and hard verbs.

### Typical and Non-typical Regulars and Irregulars

Given these results we used the activation ratio to establish “typical” and “non-typical” regular and irregular verbs from the imaging perspective. Typical regulars were regulars with a high activation ratio. The 10 most typical monosyllabic regulars according to this measure, given the specific training set of our model, were *nail, nurse, roar, hail, hiss, slice, frost, dawn, roast*, and *rate*. Note that these are not the most frequent regulars because frequent verbs also highly activated the indirect pathway, leading to a lower activation ratio. The 10 least typical monosyllabic regulars, that is, those regulars with the lowest activation ratio, were *ask, look, roam, mask, add, try, soil, dry, hum*, and *use*. Interestingly, several of these verbs would normally be regarded as prototypical regulars because of their high frequencies. The synthetic imaging results presented here, however, predict that in brain imaging studies they might actually activate similar regions to irregulars.

The 10 irregular verbs with the most typical irregular activation pattern, that is, a low activation ratio, were *say, see, think, stand, bring, do, go, make, get*, and *speak*. The 10 least typical irregulars according to this measure were *shrink, spin, sweep, flee, deal, creep, thrust, kneel, ride*, and *quit. Five* of these 10 verbs (*sweep, flee, deal, creep*, and *kneel*) are pseudo-regulars which add [t] or [d] to their past tense and, according to Joanisse and Seidenberg (2005), should be expected to cluster with regular verbs in their activation profile. In line with this claim, these verbs showed “regular-like” activation patterns in the NCM.

### Analysis From a Dual-Mechanism Perspective

Although the NCM shows that regional double dissociations between regular and irregular verbs can emerge solely on the basis of the statistical properties of different verbs in a single processing mechanism that is shaped by experience-developmental structural development, and that the grammatical property of regularity plays no role in causing these dissociations, in brain imaging studies the underlying mechanisms remain unknown and are hypothesized on the basis of observed data. Thus, when dissociations between regular and irregular verbs are observed in an empirical study, researchers adopting a dual-mechanism framework explain these data in terms of separate processing mechanisms (Jaeger et al., 1996; Lavric et al., 2001; Beretta et al., 2003; Dhond et al., 2003; Sahin et al., 2006; Oh et al., 2011), with regions more active for regulars hypothesized to be responsible for the application of grammatical rules, such as regular inflection, and regions more active for irregulars indicating the retrieval of full forms from the mental lexicon located in this region. At the core of such dual-mechanism interpretations lies the assumption that grammatical class (i.e., regularity) forms the basis of observed dissociations.

To mimic this inferential process from data to hypothesized mechanism, we analyzed the activation differences in the model from a dual-mechanism perspective, which would assume that regularity itself is a predictor of the observed dissociations. We performed further multi-level regression analyses for pathway activation, with verb and network as random effects, and with regularity added to the inventory of independent variables first modeled in section Origins of Dissociations (each zero centered and scaled, *SD* = 1). Results were again highly significant, with past tense frequency (β = 15.35), regularity (β = −36.28), complexity (β = −4.65), and friends (β = −1.68; all *p* < 0.001) predicting indirect pathway activation (*R*^2^ = 0.557). This model accounted for ~6% more variance than the model without regularity as a factor, and this increase was significant (*p* < 0.001). Including regularity as a predictor in the regression model of direct pathway activation did not lead to a statistically significant improvement in model fit, and regularity did not predict activation (*p* = 0.638).

Although the NCM is a single-mechanism model, the results for indirect pathway activation correspond to the predictions made by the revised version of the dual-mechanism theory for lexical retrieval (Pinker and Ullman, 2002). This revised theory predicts lexical retrieval not only for irregulars but also for high frequency regulars because high frequency forms are more likely to be memorized than low frequency ones, and for regulars with low friend-enemy ratios because they are more likely to be attracted to irregular enemies. From a dual-mechanism perspective, activation patterns like those observed in the model would therefore be taken as backing for this theory, despite being caused by a very different underlying mechanism. This result highlights the benefit of computational modeling: when we collect empirical data we do not know the mechanism that generates them but we infer from the data to a potential underlying mechanism. When we construct a model we know the mechanism and we see how this mechanisms generates empirical data. In the past tense debate, empirically observed dissociations between regulars and irregulars have often been hypothesized as arising from two separate underlying mechanisms. The NCM shows that such dissociations, down to a level of detail that has previously served as refinement of the dual-mechanisms theory, arise in a single-mechanism system on the basis of statistical properties of verbs together with experience-dependent structural development. By designing the model we know that whether a verb is regular or not is not encoded in the training data and is therefore not accessible to the network. The fact that regularity nevertheless emerges as a significant predictor for indirect pathway activation is a consequence of two factors: on the one hand regularity correlates highly with several of the measures that lead to specialization of the network pathways (Table 2). Regular verbs tend to be less frequent, have more friends and fewer enemies than irregulars, and are phonologically less complex. As discussed above, learning high frequency verbs with few friends and many enemies leads to the allocation of hidden units in the indirect pathway, and thus to higher indirect pathway activation in the adult model. Therefore, regular verbs, which show the opposite profile, will on average have fewer dedicated hidden units and thus a lower activation of the indirect pathway, with these forms activating the direct pathway more.

**Table 2:** Correlations between regularity and the statistical properties of verbs in the training data.

**Correlation with regularity**	**Past tense frequency**	**Friends**	**Enemies**	**Phonological complexity**
*r*	−0.363	0.176	−0.7	−0.122

*All correlations p < 0.001*.

Nevertheless, this cannot be the sole explanation of the significant effect for regularity, because if regularity was entirely predictable from these factors the hierarchical regression should not show a significant improvement when regularity is added. Instead, the explanation lies in the fact that associative learning mechanisms make use of all cues that facilitate learning of a mapping. In the case of the English past tense, these cues do not only lie in the distributional characteristics of verbs available to the model indirectly through the training schedule, but also in the phonological characteristics of verbs, which are available directly as inputs. The second factor explaining why regularity emerges as a significant predictor of activation in the indirect pathway is that the mapping between stem and past tense for regulars is easy to learn in the direct pathway because regular verbs have the highest relative and absolute type frequency. To form their past tense, in English more verbs preserve their stem and add –*ed* than undergo any other transformation. Therefore, additional structure in the hidden pathway is not necessary for learning this transformation. As such, regularity-specific activation patterns in the model arise out of a combination between structural and distributional environmental cues together with the model's experience-dependent developmental process.

### Mapping the Model to the Brain

A question that can be addressed in SBI studies is which brain areas give rise to specific behaviors. Indeed, one way in which SBI has been applied is in modeling the internal functioning of, and the interactions between, specific brain areas that are known to be involved in a task. For example, the model developed by Cangelosi and Parisi (2004) contained a sensory layer and a sensory/proprioceptive layer, and results from processing nouns and verbs were linked to previous fMRI results showing that nouns activate more sensory areas and verbs activated motor areas. Similarly, Horwitz et al. (1999) presented a biologically plausible large scale neural model of the interactions between specific brain areas, and used this model to account for cerebral blood flow data from PET studies. Nevertheless, of course, even “biologically plausible” models are far away from the biological substrate of the brain in terms of detail and number of interacting regions. Any link between activated regions in a higher-level model and the brain can therefore only serve as a suggestion that should be verified in subsequent neuroimaging studies. As such, speculating on such links can be beneficial for generating predictions about which stimuli might activate which brain areas preferentially.

Can a mapping between model and brain areas also be done on the basis of the present past tense model? One difficulty is that by necessity, the model is simple and the brain is complex, making any such attempted link seem tenuous. On the other hand, though, as the model clearly develops specialized processing pathways, it might seem a missed opportunity not to at least speculate how the model's pathways might map onto brain structures. A second difficulty in attempting such a mapping is that, unlike in the models described above, the data from past tense imaging experiments are anything but clear. As discussed above, studies have differed greatly in the areas that were found to be involved in regular and irregular processing. Furthermore, most imaging studies were not principally concerned with testing whether specific brain areas were involved in inflection processing, but instead investigated whether regular and irregular verbs activated different brain areas in principle (to provide evidence for dual-mechanism accounts of inflection) or whether regions for regulars and irregulars overlapped and dissociations were based on phonological and semantic factors (as evidence for single-mechanism accounts). When differences were found they were typically explained in a *post-hoc* manner. In one study favoring a dual-mechanism interpretation, for example, Dhond et al. (2003) noted that the left fusiform area, which was activated more by irregulars, has been implicated in lexico-iconic or word-form encoding and early lexical access, whereas Broca's area, which was in this study activated more by regular verbs, plays a role in rule-based past-tense formation, grammar, and syntactic parsing. Likewise adopting a dual-mechanism interpretation Sahin et al. (2006) and Jaeger et al. (1996) found equal activation of Broca's area for regulars and irregulars, and therefore attributed a general role in inflection processing to this region. Stronger activation for irregulars in the anterior cingulate and supplementary motor areas was in Sahin et al.'s (2006) study attributed to irregular verbs blocking the application of regular inflection, which is a central feature of dual mechanism accounts. Few studies include predictions about the specific areas involved in past tense processing. For example, Joanisse and Seidenberg (2005) hypothesized that, overall, activation should be distributed over areas responsible for phonological processing such as the inferior frontal gyrus (IFG), including Broca's area, and areas involved in semantic processing, particularly the posterior temporal lobe. These areas have also been shown in brain-damaged patients to lead to dissociations between verb types (Patterson et al., 2001; Bird et al., 2003).

The IFG and posterior temporal lobe are also a subset of the areas involved in inflection processing in Desai et al.'s (2006) study, which had the most carefully matched verb set. Given that studies with brain damaged patients also point toward these areas as being involved in inflection, we can hypothesize that the pathways in our model map to these areas. Specifically, the direct pathway in our model might reflect the functioning of the IFG, and the indirect pathway might reflect the functioning of the posterior temporal lobe. However, our model does not suggest that these are phonological and semantic areas respectively, as suggested by Joanisse and Seidenberg (2005). Instead, both reflect phonological processes, with the IFG processing direct mappings based on distributed phonological information, and posterior temporal areas providing more localist word representations that complement these distributed representations in the IFG. There has been a controversy on whether semantic representations are causal in enabling the formation of irregular past tense forms or whether semantic and irregular past tense representations are merely collocated in the IFG.

Deficits in irregular inflection are often associated with semantic impairments in Alzheimer's disease, semantic dementia and herpes simplex encephalitis (HSE) (Ullman et al., 1997; Patterson et al., 2001; Tyler et al., 2002) as a consequence to damage to the temporal lobes. Nevertheless, the association does not seem to be absolute, as would be expected if semantic representations formed the basis for irregular inflection. Several studies have reported cases in which patients with semantic deficits did not show disproportionate problems with semantic inflection (Tyler et al., 2004; Miozzo and Gordon, 2005) and others found patients with no semantic deficits but problems with irregular inflection (Miozzo, 2003). A longitudinal study of two semantic dementia patients (Bright et al., 2008) reported that the early stages of dementia were associated with semantic deficits, but that language deficits occurred later when brain atrophy was more widespread. We believe that these results raise the intriguing possibility that semantic and irregular verb representations are closely but not causally associated, because both constitute idiosyncratic representations. There is nothing in the sound of a word that signifies its meaning, and there is also nothing in the sound of a verb that predicts its irregular past tense. Both have to be learned, and it is possible that idiosyncratic information connected with words is stored in the posterior temporal areas of the brain. Computational modeling work supports this view. A well-known model of past tense impairment after brain damage (Joanisse and Seidenberg, 1999) included a set of localist units for each verb, and damage to these localist units led to greater irregular impairment. However, while these units were labeled as “semantic” in the model, there was nothing to connect them to the meaning of words; their main role was to encode idiosyncratic information. Likewise, in our neuroconstructivist model we did not include “semantic” representations (in the sense that the meaning of verbs was not encoded) because we found that a model without semantics (albeit with the ability to encode idiosyncratic information without recourse to semantics) accounted for a wide range of behavioral data in past tense tasks, spanning acquisition, adult processing and impairment after brain damage, and that assuming a causal role for semantics was therefore not necessary.

The proposed mapping of the pathways in our model to brain areas is corroborated by results from selectively damaging the model's pathways (Westermann and Ruh, 2012), where damage to the indirect pathway selectively affected irregular verbs and damage to the directed pathway affected all verbs, albeit regulars to a greater degree. These results correspond to patients with brain damage to left temporal areas and the IFG, respectively. Further evidence linking the direct pathway to the IFG comes from the fact that in Joanisse and Seidenberg's (2005) fMRI study, “pseudo-regulars” (such as *burnt*) clustered with regulars in the IFG, and the same was true in the model where several pseudo-regulars had activation ratios comparable with regular verbs. Finally, locating the direct pathway in the left IFG can provide insight into why some studies found equal activation for all verbs in Broca's area (Desai et al., 2006; Sahin et al., 2006), while others found greater activation for regular verbs in this region (Dhond et al., 2003). In our model, the direct pathway is strongly activated by all verbs, but slightly more by regulars. Whether activation differences in this path are found therefore depends on the precise choice of verbs. In accord with Joanisse and Seidenberg (2005), the model predicts that activation in the IFG is associated with phonological processing rather than with regularity: In our regression analysis of direct pathway activation, phonological complexity was the strongest predictor, but regularity was not a predictor.

## Discussion

The simulations described in this paper show how dissociations between brain activation patterns in inflection tasks can arise from a single associative mechanism together with experience-dependent structural development. The argument that dissociations between verbs reflect ease of processing has been made previously with respect to imaging studies (Seidenberg and Arnoldussen, 2003), but the present model provides a mechanistic account of how these dissociations can arise and a precise characterization of their underlying factors. Importantly, the model predicts that frequency acts in the opposite direction to ease of processing, and that hard-to-process verbs should generate similar activation patterns to high frequency verbs because dedicated structure in the developing system is allocated to both.

Together these results raise a number of important points. First, dissociations in activation patterns like those observed in the model have often been described as being between regular and irregular verbs, and have been taken as evidence for the existence of qualitatively distinct mechanisms (i.e., rule application and lexical retrieval) in the inflection of these verbs (Jaeger et al., 1996; Bergida et al., 1998; Lavric et al., 2001; Dhond et al., 2003; Sahin et al., 2006). The fact that the same dissociations emerge in the model on the basis of a single processing mechanism considerably weakens this argument. Whereas separate processing mechanisms would result in observable dissociations, the reverse implication is not true: Separate mechanisms are not *necessary* to obtain dissociations [see also Plaut (1995)]. Second, although superficially the emerging dissociations appear to be between regular and irregular verbs, their true nature is better described as a grading between low-frequency, phonologically complex verbs with many phonological friends and few enemies on the one hand, and high-frequency verbs with many enemies and few friends on the other. Although these statistical factors correlate with regularity, characterizing the dissociations observed as being between regulars and irregulars is a *post-hoc* abstraction of the actual underlying mechanisms. When this abstraction is used as an explanation of the underlying processes, as in dual mechanism approaches, a lot of the empirical data, such as the gradation of dissociations and the effects of phonology, friends, and enemies, cannot be captured. Third, regional activation patterns in imaging studies are likely to be a complex function of the statistical and phonological properties of the verbs used in a specific study. All imaging studies have taken this possibility into account and controlled for various properties. However, the selection of properties controlled for has generally not been systematic or based on evident theoretical considerations. The results presented here suggest that interactions between verb frequency, phonological complexity, and numbers of friends and enemies are the main factors affecting regional activation differences. Fourth, these results indicate that typical (i.e., high frequency) regulars and irregulars might not in fact activate different brain regions. Instead, according to the model, all frequent verbs share activated regions, and dissociations between regulars and irregulars will primarily be found among low frequency verbs. Finally, some previous approaches have also based explanations for dissociations on a differential involvement of semantics in the generation of regular and irregular forms (e.g., Joanisse and Seidenberg, 1999; Patterson et al., 2001). In this view, regular inflections rely on phonological representations, whereas irregular inflections are based on the semantic representations of verbs. Without precluding the possibility that semantic and irregular processing might be linked and correlated, the present model, which does not contain semantic representations, suggest that semantic and irregular impairments might correlate because they both refer to idiosyncratic information about verbs that cannot be directly retrieved from their phonological form.

In line with our argument that experience-dependent brain development shapes the adult cognitive architecture, the performance of the NCM model is an outcome of its experience with the learning environment. While we have made specific predictions about what are typical and atypical regular and irregular verbs in terms of brain activation patterns, as well as about the statistical factors predicting activation patterns in the model pathways, this point must serve as a caveat because it is not clear how closely the statistics of our verb set reflect those of real-world language learners. For example, the frequency statistics in our corpus are extracted from the CELEX database (Baayen et al., 1995) and are not derived from parental input to a child. Likewise, with respect to modeling, we made decisions about what statistical factors to consider in the first place. These choices were guided by two considerations. First, the factors must be available to the model. Therefore we did not include, for example, imageability as a factor because the model does not contain semantic representations. Second, a factor must be available for the majority of verbs. This precluded our use of age of acquisition norms, which are only available for a relatively small subset of verbs. Given these caveats, a worthwhile avenue for future research will be to investigate how variation in the input comes to be reflected in variation in the model's architecture and performance, and in how far model performance is robust to input variation.

In a similar vein, our model effectively isolates the past tense inflection process from the rest of cognitive processing. On the one hand this is a valuable abstraction because it allows for a precise investigation into the factors affecting activation patterns in this task alone. On the other hand, it is possible that different inflectional paradigms such as noun plurals or even inflections across languages known to multilinguals affect each other. While computational models exist that have simultaneously learned multiple inflections in a single system (Plunkett and Juola, 1999) there has been no systematic investigation of how these paradigms affect each other. Likewise, although we argued that the semantics of verbs are not causally linked to irregular inflection (Westermann and Ruh, 2012), omitting semantic representations from the model does not allow it to distinguish between homophones (e.g., *ring* and *wring*) and consequently these were excluded from the training data.

Although the model provides a precise account of the origins of different activation patterns in synthetic brain imaging, it is nevertheless possible that verbs might dissociate differently depending on the experimental paradigm, because representations in different areas for the same verb might be redundant. As discussed, the model predicts that in imaging studies inflecting frequent and hard verbs activate the same brain regions. It is, however, possible that in behavioral paradigms such as lexical decision tasks, frequent and hard verbs might dissociate as interactions between processing regions can differ with specific task demands even when the same regions are involved in processing both. This is because in a neuroconstructivist system that structurally develops on the basis of experience with the environment, a brain area that is activated by a certain process need not be necessary for this process because such a system would involve a degree of redundancy. For example, although high frequency verbs in the model activated the indirect pathway more than low frequency verbs, this does not mean that the indirect pathway is *necessary* for the production of high frequency past tense forms. Instead, their production might be possible based on the direct pathway alone, with indirect pathway representations being redundant. This would also indicate that one could expect differences between results from brain imaging and from behavioral studies with brain damaged patients (Price and Friston, 2002; Thomas et al., 2012). Damage to a certain brain area would therefore affect forms that activate this area in different ways, depending on whether the area is redundant for the processing of a specific form or not. For example, as described above, in lesioning the NCM to simulate selective impairment after brain damage (Westermann and Ruh, 2012), even when the indirect pathway was completely lesioned performance on regulars remained virtually unimpaired, indicating that the direct pathway is sufficient for producing all regular forms, despite, as reported here, regulars also activating the indirect pathway in synthetic brain imaging.

As a more general contribution, the model presented here highlights the importance of computational modeling in understanding the mechanisms of cognitive processing. As shown in the regression analyses, depending on the adopted theoretical perspective different explanations can be derived from the same observed dissociations. Under the assumption that the grammatical class (i.e., regular or irregular) of a verb is accessible to the model and analyzing the observed activation patterns from this perspective, the results would be taken as evidence for a dual-mechanism view of inflection processing. In studying the brain, this top-down approach from observed data to potential underlying mechanisms is the only possible approach. In a computational model, however, the mechanisms of processing are known and we can observe what data is generated through these known mechanisms. Using this bottom-up approach we know that regularity is not one of the factors accessible to the model, and that all inflections are based on a single mechanism that operates in a structured processing system. Likewise, the model has no access to semantic representations, with all inflections based on phonological information alone. Finding that dissociations between regular and irregular verbs nevertheless emerge under these constraints disconfirms the claim that such dissociations are evidence against a single-mechanism explanation and necessitate a dual-process system. However, these results also weaken the argument of prior single-mechanism accounts that semantic representations play a causal role in the inflection of irregular verbs. Computational modeling provides a detailed alternative explanation to these views by quantifying the interactions between statistical verb properties that give rise to the observed dissociations, and by providing a mechanism by which the structure of the environment comes to be reflected in the structure of the processing system through neuroconstrucivist development. Computational modeling is therefore an important approach in the gathering of converging evidence for theories of inflection processing, and for theories of cognitive processing in general.

Finally, together with previous work incorporating the NCM (Westermann and Ruh, 2012), which accounted for empirical data from past tense acquisition, adult generalization, and impaired processing after brain damage, we believe that our modeling of brain imaging data in the current paper illustrates how neuroconstructivist computational modeling can overcome one point of criticism sometimes levied against models in this domain—that each individual model is tailored specifically to account for a single phenomenon (Pinker and Ullman, 2003)—in providing a principled account of past tense processing by explaining existing data as well as generating predictions for future research.

## Data Availability Statement

The datasets presented in this study can be found in online repositories. The names of the repository/repositories and accession number(s) can be found at: Open Science Framework, https://osf.io/ejs4m/.

## Author Contributions

GW developed the study hypothesis, ran the simulations, and drafted the paper. GW and SJ performed the data analysis, discussed the results, and finalized the manuscript for submission. Both authors contributed to the article and approved the submitted version.

## Conflict of Interest

The authors declare that the research was conducted in the absence of any commercial or financial relationships that could be construed as a potential conflict of interest.
